# The Lived Experiences of Community Health Workers Serving in a Large-Scale Water, Sanitation, and Hygiene Intervention Trial in Rural Bangladesh

**DOI:** 10.3390/ijerph18073389

**Published:** 2021-03-25

**Authors:** Mahbubur Rahman, Tania Jahir, Farzana Yeasmin, Farzana Begum, Mosammot Mobashara, Khobair Hossain, Rizwana Khan, Rezwana Hossain, Fosiul Alam Nizame, Anika Jain, Elli Leontsini, Leanne Unicomb, Stephen P. Luby, Peter J. Winch

**Affiliations:** 1 Infectious Diseases Division, International Center for Diarrheal Disease Research, Bangladesh (icddr,b), Mohakhali, Dhaka 1212, Bangladesh; tania.jahir@icddrb.org (T.J.); fyeasmin@icddrb.org (F.Y.); farzanab@icddrb.org (F.B.); ruma.aditee@gmail.com (M.M.); khobairbd@gmail.com (K.H.); rizwana.khan@icddrb.org (R.K.); 13rezwana@gmail.com (R.H.); fosiul@icddrb.org (F.A.N.); leanne.unicomb60@gmail.com (L.U.); 2 Department of International Health, John Hopkins Bloomberg School of Public Health, Johns Hopkins University, Baltimore, MD 21205, USA; anikajain@gmail.com (A.J.); eleontsi@jhu.edu (E.L.); pwinch@jhu.edu (P.J.W.); 3 Division of Infectious Diseases and Geographic Medicine, Stanford University, Stanford, CA 94305, USA; sluby@stanford.edu

**Keywords:** community health workers (CHW), CHW interventions, WASH, incentives, recruitment, retention factors

## Abstract

Community health workers (CHWs) are key to implementing community-based health interventions and quality can be enhanced by better understanding their lived experiences. The WASH Benefits, Bangladesh trial engaged 540 female CHWs to promote varying health intervention packages. We report on factors influencing their lived experiences during the trial, to aid future recruitment, training and retention of CHWs. Nine focus groups and 18 in-depth interviews were conducted with CHWs. Focus groups and interviews were transcribed and thematic content analysis performed to summarize the results. All CHWs described experiencing positive working conditions and many benefits both socially and financially; these contributed to their retention and job satisfaction. Their honorarium was commonly applied towards their children’s education and invested for income generation. CHWs gained self-confidence as women, to move unaccompanied in the community and speak in public. They earned respect from the community and their family members who helped them manage their family obligations during work and were viewed as a resource for advice on health and social issues. Many participated in family decision-making from which they were previously excluded. Health programs should foster a positive experience among their CHWs to aid the recruitment, retention and development of this important human resource.

## 1. Introduction

Community health workers (CHWs) are essential in implementing community-based health interventions and promoting preventive behaviors. The term CHW refers to all cadres of community-based workers including community health promoters, community health advocates, community outreach workers, lady health workers, community health volunteers, village health workers and auxiliary health workers [1,2]. Despite the varying terminology, their descriptions and roles are generally similar in that they provide services to promote a healthy lifestyle and prevent diseases, mobilize and encourage community members to utilize health services and facilitate access to facility-based healthcare, as noted by a systematic review [3]. The World Health Organization defines CHWs as “*members of the communities where they work, selected by the community, answerable to the communities for their activities, supported by the health system but not necessarily a part of its organization, and have shorter training than professional workers*” [2].

In many low- and middle-income countries (LMICs), there are critical shortages of highly educated health professionals [4]. CHWs’ ability to make efficient use of the limited resources available and shift into different roles is instrumental in ultimately improving the health of millions at a reasonable cost [5]. A systematic review revealed that CHWs improved participant knowledge, brought about greater positive behavioral changes and health outcomes in some cases and reduced costs of maternal and child health interventions, when compared to alternative learning approaches [6]. There have been many instances of rigorously demonstrated improvements in health outcomes utilizing CHWs [7,8,9].

Globally, CHWs are overwhelmingly female [10]. Like other South Asian countries, Bangladesh has a male-dominated society where females are confined in the domestic domain, by religious tradition, social norms and cultural restrictions [11]. For Bangladeshi females, traveling alone freely or working outside the home is generally socially unacceptable, while it is the males who are responsible for decision making, handling financial issues and representing the household in the outside world [11]. However, beyond their responsibilities in the home, Bangladeshi women, even those with limited education, are increasingly engaged in paid work outside the home, including the garment industry and the health sector. There are domains within the health sector such as reproductive health, where contributions by female health workers are particularly necessary for effective promotion [12]. Thus, because female CHWs work within patriarchal societies, the role they play to promote public health, can potentially empower them, both financially and socially [13].

CHWs’ effectiveness depends on their level of motivation which in turn is influenced by both extrinsic and intrinsic factors [14]. A study conducted in Bangladesh examined how CHW retention or attrition is influenced by their job satisfaction and their community’s valuation. Job satisfaction included feasibility of workload, incentives, costs, supervision, and operational support [15]. CHWs need incentives to be recruited, retained and implement interventions effectively and sustainably [14]. While monetary incentives are important, non-monetary incentives including personal development, training, support networks and building relationships with the community are also valuable for CHWs [14]. Because many health programs rely on the service of CHWs, it is worth exploring factors affecting the CHWs’ lived experiences while serving in the program. These factors, in turn, can be leveraged when recruiting, training and retaining CHWs, to have a positive impact on the social and economic status of CHWs.

Efforts to improve the utilization of CHWs in health projects are hindered by a lack of understanding of sources of extrinsic and intrinsic motivation and the impact that being a CHW has on their lives. For example, a study conducted in rural Nepal examined how part-time employment with a small income affected local women who were hired to assist with a nutritional intervention project [16]. The study analyzed the household food expenditure and nutritional status of those women who were hired compared to women who were not. After two years, there was no significant difference in nutritional status, and the changes in food expenditure were similar between the women who were hired for the project and those who were not [16]. That study did not explore the project’s effects on household expenditure beyond food nor the social effects within the household or community. Thus, the CHWs’ full experience often remains under-studied. Along with other criteria, a well-balanced examination of social acceptance of CHWs and their work is a prerequisite for their effective deployment in the health sector.

In this paper, we present findings on the lived experiences of female CHWs serving in the WASH-Benefits Bangladesh trial to inform how future community-based health interventions should employ, support and supervise CHWs.

## 2. Materials and Methods

### 2.1. Study Setting and Study Population

The study was nested within the WASH Benefits study, one of the largest cluster-randomized trials (ClinicalTrials.gov NCC01590095) in the WASH sector with the following cluster assignments: (a) drinking water treatment and safe storage (10 L vessel with a lid and tap and monthly supply of sodium dichloroisocyanurate (NaDCC)) (b) sanitation (double pit latrine, sani-scoop and potty) (c) handwashing (two sets of handwashing stations, soapy water bottle and detergent sachet) (d) nutrition supplementation (lipid-based nutrient supplement, Nutriset, Malaunay, France) promoting exclusive breastfeeding and complementary feeding, as per national guidelines (e) combined water and sanitation plus handwashing (WSH) (f) combined nutrition and WSH and (g) non-intervention control group [17,18].

Clusters were randomly allocated to each intervention arm, pair-matched by geography, with adjacent clusters randomized in blocks. Each cluster was a group of compounds visited by a single local CHW and separated by at least a 15 min walk [17]. Clusters and their allocation were set in this way in order for the WASH Benefits study to generate rigorous evidence for the impact of water, sanitation, hygiene, and nutrition interventions alone or in combination on child health and development, based on the participants’ high behavioral uptake achieved by the trial’s female CHWs [19].

CHWs studied here were the main workforce to disseminate the behavior change interventions, and they came from the same four districts of Bangladesh as the participants of the WASH Benefits study: Gazipur, Kishoreganj, Mymensingh and Tangail.

### 2.2. Recruitment, Training, and Supervision of CHWs

**Figure 1:**
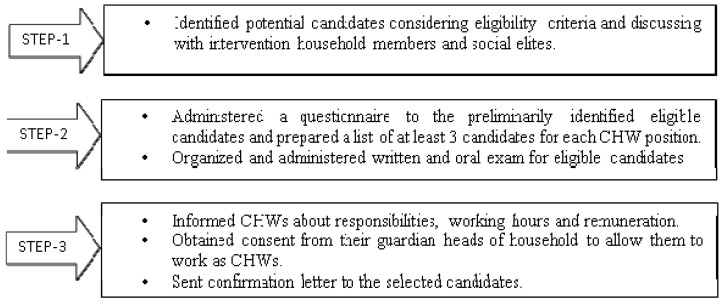
CHW recruitment process by intervention delivery study team.

We carried out the recruitment process following transparent, merit-based selection methods outlined in Figure 1. A total of 540 women who had at least eight years of education, within the age of 18–40 years and had passed a written exam and in-person interview, were recruited as CHWs to carry out the intervention activities. The CHWs were recruited as part-time workers to work four days a week for approximately four hours a day. They were offered a monthly honorarium of 1500 BDT per month (equivalent to USD 20), as well as transportation and training allowances. The study team provided training to the CHW supervisors first, who then trained the CHWs under their supervision using an interactive problem-solving approach. Each supervisor oversaw 12 CHWs. In addition to receiving on-the-job training, CHWs received a day-long refresher training every month until the intervention was phased out. During training, each CHW also received a set of commodities (a handwashing station, a water storage container, water purifier tablets and a dual pit latrine) for their own use, similar to the enabling technologies that they later distributed to the study participants. They also received a bag and umbrella for their daily use, and refreshments when attending meetings. A more detailed description of the CHW training and supervision is provided by Unicomb et al. [20].

### 2.3. The Role of CHWs in Intervention Dissemination

Each CHW carried out the intervention activities in one WASH Benefits trial cluster of 6–8 households. CHWs distributed the enabling technologies to the households or compounds corresponding to their intervention arm and conducted group education in compound-based meetings on technology use and maintenance. They followed up with frequent home visits for motivational counseling and problem solving. More detailed descriptions of the CHWs’ role in intervention dissemination are provided by Luby et al. and Parvez et al. [18,19]. Based on a monthly fidelity assessment conducted during the trial, after the first few months there was a high uptake for all the intervention arms, with sanitation having the highest uptake of 94–97% [19].

### 2.4. Study Design

After the third intervention year, between October to December 2015, we conducted a qualitative assessment of CHWs’ lived experiences while serving in the trial, guided by CHW recruitment and retention conceptual frameworks [14,15]. We chose a qualitative research design in order to study in-depth the lived experiences of the implementers.

Skilled qualitative interviewers conducted nine focus group discussions and 18 in-depth interviews with a total of 135 CHWs. Average number of participants per focus group was 13. We purposively selected participants for in-depth interviews with consideration of CHWs’ working area, assigned intervention arm and performance (Table 1). Supervisors periodically observed CHWs’ activity and recorded their observation on a form, in order to evaluate their performance and target their mentoring accordingly. Based on these observations, supervisors ranked CHWs into three categories: ‘A’ as high performing, ‘B’ for average performing or ‘C’ for poor performing, considering the following criteria:

o CHW’s ability to use behavioral change communication materials such as flipchart que-cards
o CHW’s message delivery skill: how they interacted with mothers and how they responded to the mothers’ queries
o Punctuality: conducting courtyard meetings/visiting households on time
o Ability of solving problems emerging at field level
o Their seriousness in performing assigned tasks

**Table 1:** Distribution of CHW participants by assigned intervention arm and data collection method.

Type of Participants (*N* = 135)	Water (W)	Sanitation (S)	Hygiene (H)	Nutrition (N)	WSH	WSH + N	Total
Participants for in-depth interviews	3	3	3	3	3	3	18
Participants for 9 focus groups	21	20	20	18	20	18	117

For in-depth interviews, we selected participants from each category of performance, proportional to number of CHWs in that category: we conducted 11 in-depth interviews from category ‘A’ because the highest number of CHWs were ranked in category “A”; we interviewed six from category ‘B’ and one from category ‘C’. For focus groups, as the geographic intervention area was divided into nine sectors of 80 clusters each, we conducted one focus group in each sector. When issuing focus group invitations to the CHWs, we considered participants from all intervention arms and all performance categories. As the focus groups were conducted in the field office, we excluded participants who lived in distant locations, had young children, or were unable to travel to the field office.

### 2.5. Data Collection and Analysis

The data collection team included anthropologists and social science graduates who were well trained and experienced in conducting qualitative research. Guidelines for in-depth interviews and focus groups were developed based on study objectives guided by recruitment and retention determinants identified in the literature, such as motivation for becoming a CHW, the role of training, gender, working outside the home, familial opposition, economic status, community valuation, workload, monetary and non-monetary incentives such as supportive supervision, identification badge, appropriate job aids and other materials necessary for the CHW to conduct their job well, and fulfilment of pre-hire expectations [14,15]. These are examples of questions included in the data collection tools:

(1) What are the most important material resources that you need to succeed as a CHW and why are they necessary? Did you feel like you had adequate access to the material resources you needed to do your job well? What other resources do you think you needed?
(2) How, if at all, have you changed while working as a CHW? What do you think caused those changes: icddr,b (icddr,b is the brand name of the International Center for Diarrheal Disease Research, Bangladesh.), community, relationships with households, family relationships, contact with other CHWs?
(3) Has the honorarium paid by icddr,b brought any changes to your financial condition? In what way? Were you satisfied with the amount you were paid and the other benefits you received from icddr,b? Why or why not?
(4) Did you feel any changes in your life after being engaged as a CHW in WASH-B study? What are those? How? (Probe for: Monetary contributions, Changes in status such as participating in the decision-making process in the family, Empowerment such as confidence to talk publicly, mobility, identity, reputation, social acceptance)

On an average, each of the in-depth interview took 50 min and focus group discussion took 90 min. In-depth interviews involved a single researcher while focus groups were conducted by a main facilitator accompanied by a co-facilitator/note taker. Researchers recorded all in-depth interviews and focus group discussions with digital audio recorders and took notes during the group discussions. All recorded data were transcribed separately in the native Bengali language. The research team then prepared a thematic code list according to the topics in the data collection tools and coded each transcript based on the predefined themes, as well as new themes emerging from the data [21]. There were three coders in total. In general, there was high agreement among coders because the study themes were non-controversial. Whenever there was a coding doubt, the coder brought it up for discussion among all coders and agreement was reached by consensus. The coded textual data were then translated and summarized in English by thematic content and presented back to the CHWs during continuing work meetings, for member checking [22]. Direct quotes from the transcripts were included in the data summaries to illustrate each theme for the CHWs and future readers and ensure the accuracy of the analysis [23].

### 2.6. Ethical Considerations

The data collection team read the consent form to participants and obtained their written informed consent. Verbal consent was also obtained before recording any data using the audio recorder. The study protocol was approved by the Institutional Review Board at the International Center for Diarrheal Disease Research, Bangladesh (PR11063), the Committee for the Protection of Human Subjects at the University of California, Berkeley (2011-09-3652), and the institutional review board at Stanford University (25863).

## 3. Results

### 3.1. Demographic Characteristics of the Study Participants

Table 2 displays the participating CHWs’ demographic characteristics. Their mean age was 29 and most (96%) were married. Among them 16% had completed 12 years of schooling, half (50%) had completed 10 years of schooling, and 17% had completed 8 years of schooling. Most (75%) of the CHWs were working in the trial for 2 to 3 years. Over the study period 7% of the CHWs left the job due to marriage, migration, sickness, and childbirth; we replaced them as they left the job.

### 3.2. Emerging Topics and Themes

The findings from the interviews and focus groups conducted with the CHWs can be broadly categorized under four major topics listed in Table 3: (1) anticipated benefits in becoming a CHW (2) benefits experienced while serving as a CHW (3) challenges connected with CHW service and (4) perceived profile of a successful CHW. During thematic analysis six major themes emerged, described below and summarized in Table 3, along with their definitions.

#### 3.2.1. Anticipated Benefits of Becoming a CHW

##### Theme 1: Importance of Financial and Other Factors Influencing Decision to Become a CHW

Though this assessment took place after the 3rd year of intervention, CHWs remembered their expectations when they first heard about the opportunity to work for the project. They were eager to be recruited as a CHW for various reasons. Most (16/18) of the in-depth interview participants and many in the focus groups mentioned their expectation to receive an honorarium or financial incentive. They anticipated that this money would help them fund their personal needs, contribute to family expenses and cover their own educational expenses.

One 28-year-old married in-depth-interview respondent said:

*My husband forbade me to continue my studies as I was pregnant. Then I thought, if I joined as a CHW, I could bear my study expenses with the honorarium, and I could continue my graduate study.*

Another CHW stated:

*If I could earn 1500 BDT per month then I will be able to face any financial problem easily. If I don’t work, then no one will help me. Also, I can spend money as per my wish. If I am not working, then I cannot do so.*

Other reasons mentioned for applying to serve as a CHW were to acquire knowledge on hygiene and nutrition to rear their own children, earn respect from the community, remove the label of being unemployed, serve society, gain experience for their resume to help them get a job in the future, gain confidence and reduce reticence to talk in a public forum.

Some said that they joined as CHWs to associate themselves with icddr,b, a well-known and reputable institution that served the people of Bangladesh. In this regard, one of our in-depth interview participants mentioned:

*I knew that icddr,b is a well-known and renowned organization that works on health-related issues. It invented oral saline and millions of lives are saved with it.*

#### 3.2.2. Benefits Experienced While Serving as a CHW

##### Theme 2: Increased Knowledge and Skills

All participants reported that after attending the study training sessions, their knowledge on hygiene and nutrition increased. They explained that before attending these sessions they knew very little and did not practice the recommended behaviors. They learned to work as a skilled CHW, which involved developing the ability to respond to questions on WASH-related issues, conduct courtyard and community meetings and deliver behavior change interventions. They gained knowledge about the correct steps for handwashing with soap, information about safe and unsafe water, the names of at least five water-borne diseases, the role of maternal and child nutrition in childbirth and child growth, how to improve sanitation and how to repair hardware. As women, their involvement as a CHW allowed them to move freely outside their home environment. They also gained the ability to talk confidently in a public forum. Their job responsibilities led them to acquire the skill of building rapport with new people and then motivating and convincing them to change their behaviors. As one participant explained:

*Each lesson and every technique that I learned from these training sessions is new for me, as I knew almost nothing about water, hygiene, sanitation and nutrition. I was also an amateur to go out and work with unknown people from outside my home.*

##### Theme 3: Family and Community Valuation

Participants were asked what their families and neighbors’ first impressions were of them working as a CHW, and in most cases, they replied that they received positive responses and attitude from them. This was reflected through their quotes:

*My neighbors are also very nice to me. As the cholera hospital (icddr,b is locally known as cholera hospital) is well known to the village, when they came to know that I work for this hospital, they took it very positively.*

*Some of my neighbors asked for latrine and handwashing buckets; they asked me how they can get these items or how other pregnant women can be enrolled in this study.*

However, in a few cases, family members and neighbors were not supportive because working as a female CHW required talking and working with male persons while travelling around the village. After hearing about the honorarium amount, many community members undervalued the CHW position and job responsibilities, at least initially.

One interview respondent said:

*I tell them that I have been working for icddr,b in WASH study. After knowing my honorarium, they told me that I should not work for the little amount of 1500 taka. If I stay at home, then no one is going to pay me this amount of money. In case of my need, no one will help me. At the emergency moment, 1500 taka is worth 15000 takas to me. This job was like a dream come true for me. I never thought that I could work. And I am satisfied with the amount. Now they understand the situation.*

Other CHWs reported that community residents were not convinced that the enlisted pregnant mothers were randomly selected, and all of them demanded that they also receive WASH hardware distributed to study participants in the intervention arms, such as handwashing stations. One focus group participant explained that:

*Although I faced lots of problems, I was not frustrated.*

However, participants reported that the negative attitudes of their families and neighbors changed with time, and as people noticed the changes in their health status and the surrounding environment. Members of the community found that CHWs were well behaved, and they performed their job sincerely. They gained their community’s trust because CHWs performed every task which they were committed to do. When collecting data for this study, after three years of delivering the intervention, CHWs reported that community residents appreciated the project activities and wanted the project to continue. Many pregnant women expressed their willingness to be enrolled in the WASH Benefits project and many members of the community expressed their willingness to work as CHWs.

All participants frequently admitted that their experiences of working as a CHW changed their lives over time. They mentioned how their social status, financial capability and influence in family decision making increased over time.

One CHW shared this experience:

*After being a CHW, my family members show me a different type of respect. They might consider that I should have some knowledge and skill to prove myself as a working woman.*

Another shared that:

*This year my parents sent me, and I attended the parents’ meeting in school as guardian for my younger sister, because my parents consider me as more knowledgeable and trust me.*

Working as a CHW made them well known in the community and earned them respect. One CHW reported:

*One of my enrolled study mothers sought a suggestion from me as to which school she should get her elder son admitted. She trusts and respects me so much.*

As the WASH Benefits study progressed for three years, participants said it provided them the opportunity to bring positive changes to the status of the CHWs in the community and earn trust from the community residents.

##### Theme 4: Importance of Monetary and Other Incentives

Most participants mentioned that practicing the promoted behavior helped them to share their own experiences with household members to foster behavior change. Both focus group and interview participants mentioned that obtaining intervention hardware was an incentive. The following quotes from the CHWs during focus group and interview sessions illustrate this point:

*If I had not received this latrine and handwashing bucket people would have doubted my work. They would tell me, why you are advising us? you are not using those things!*

*As I personally use this and have experience with it, I can easily motivate people about this. Through my experience I could notice whether index mothers are using these or not.*

*When enrolled mothers come to my house and see my latrine, handwashing bucket and water container are well maintained they also get motivated to do the same.*

They also mentioned the material benefits of serving as CHWs (e.g., transport, training received, and program materials including a bag, umbrella, notebook, pen, and CHW guide) were an extrinsic incentive to continue conducting their job as CHWs.

Most (16/18) respondents mentioned that they experienced monetary benefits from participating in the WASH Benefits trial as CHWs. The honorarium was commonly used to offset the cost of children’s education (7/18 in interview and 13/117 in the focus group), utility bills, and house construction. One participant explained:

*I have two kids. My husband pays for the educational expenses for one of them, and I pay for the other. If I would not earn this money, it would be very hard for us to pay for their expenses.*

Another one reported:

*I bought a rickshaw for my husband with two-month salary and loan. Then paid the loan from my salary.*

Six respondents from the in-depth interviews and 11 respondents from the focus group discussions mentioned that they purchased cattle, poultry or sewing machines for income generation.

One interview respondent mentioned:

*Now I can take any financial attempt courageously, bearing in mind my salary. My in-laws respect me, and I take the decision on any financial attempt.*

#### 3.2.3. Challenges Connected with CHW Service

##### Theme 5: Overcoming Anticipated and Experienced Challenges

CHWs were asked about challenges they anticipated before joining the job, and about those faced during their performance as CHWs. Six interview participants had not anticipated any challenge or difficulty in becoming CHWs. One of them mentioned:

*I did not think of any challenges because both my parents and in-law’s family supported my decision to work as a CHW.*

On the other hand, most other participants from both the focus groups and interviews had anticipated as well as experienced several difficulties throughout the period they served as CHW. Difficulties mentioned by CHWs included lack of confidence to talk in public and travel around the village alone, inability to solve problems, obstacles created by non-supportive relatives and neighbors and lack of efficiency managing family and professional responsibilities. However, over time, with building up experience, the support of the study team, and the support of changed family members, CHWs could successfully overcome all the difficulties. Regarding the ability to solve problems in the community, one CHW stated:

*Usually, whenever I faced any problem during working the community, at first, I made a phone call to my supervisor, stated the problem and sought his suggestions. I did exactly what He suggested. Sometimes He came over to solve it on the spot, sometimes he told the solutions over the phone. With the course of time, I got experienced in performing my duties and I became able to solve problems by my own. Whatever the situation is, I keep informing my supervisor.*

Regarding the CHWs’ efficiency in managing ther family and professional responsibilities, participants stated that with time they established their credibility among their family members so the latter came to value their job and look after their children and home on their behalf, while CHWs were away working.

#### 3.2.4. Perceived Profile of a Successful CHW

##### Theme 6. Important Qualities and Other Factors

In response to a question related to the qualities of a successful CHW, most participants reported that knowledge about project activities, being well mannered with people and maintaining patience while talking to community residents, were contributing factors. Some of them reported that being well equipped with the appropriate supplies and materials was another quality to successfully perform their assigned duties. Recognition from the employer organization is another quality. One CHW reported,

*ID card is very helpful to continue my job. People respect me, seeing this card hanging on my neck. Besides, it also symbolized that now, I’m in official duty.*

They also mentioned following official rules, respecting the instructions of supervisors and following them accordingly and maintaining punctuality while performing job responsibilities as contributing factors to becoming successful in their job. Recruiting CHWs with adequate educational qualification was a factor that contributed to successful duty performance. Furthermore, several reported that the skills to be successful are gained over the time. One CHW explained,

*I became skilled over the time. At the very early stage, I felt uneasy while delivering messages, felt nervous while conducting a courtyard meeting. But gradually I overcame those and could deliver my best skill at the field level.*

Lastly, CHWs reported that beyond their own qualities, easy access to help from their supervisors, timely delivery of required commodities and prompt solutions of field problems by project management also contributed to their success.

## 4. Discussion

This qualitative assessment opened a window into the lived experiences of CHWs while serving in the WASH Benefits trial. There were several effects on the social lives of the CHWs after delivering the intervention for three years. When the intervention began, most participants reported that community members were supportive of their new job and responsibilities, while a few participants had family members and neighbors who considered their job negatively and criticized their movement around the community that was part of their job responsibility. Reported factors contributing to CHW attrition in other contexts have included heavy workload, working outside their own home area, night visits, familial conflict and disappointment with pay [15]. In the early stage of the WASH Benefits trial, some CHWs experienced a lack of support from their own families for various reasons including moving around the village and working with a male supervisor, but this was reversed later.

The WASH Benefits study was successfully implemented over three years, which is a longer period than some other development projects where rural women have worked as CHWs. This long duration helped CHWs earn respect from the community and their own family as well. In rural households, one or two members engage in wage labor, and the remaining household members are dependent on these earnings. As a result, the income-earning household member gained extra respect, and became a more influential decision maker in the family. Serving as a CHW for almost three years gave those rural women respect comparable to a wage-earner in the family and contributed to their empowerment. They were asked for their opinion in family decision making, an uncommon occurrence in this rural context [24,25].

We found that earning an honorarium was a central motivation for becoming a CHW. The honorarium helped offset the costs of their own education and provided them with greater financial autonomy. These funds allowed CHWs to contribute to household and child expenditures, as well as invest in income-generating schemes. Studies have shown that a small allowance, set to cover approximately 16 h/week at a day laborer’s rate, can work as a reward comparable to that of salaried government employees [14,26]. Others report a debate on whether community health workers should be paid or not [26,27]. Though the honorarium amount in our contextual setting was modest, rural women made effective use of it. Another study found that providing incentives to the CHWs improved knowledge transmission to households because they could perform their responsibilities more efficiently [28].

Non-monetary benefits of service as CHWs included enhanced confidence, added value to livelihoods and feeling empowered in their family and in the community. This remains important in patriarchal societies where women who might otherwise be marginalized, are able to engage in high value activities that improve the health and well-being of their communities [29]. This study also suggests that providing CHWs with the same WASH enabling technologies as the community members, built confidence and credibility in their delivery of the intervention, as well as teaching by example. Community members in the study sites were more likely to respect them and be open to their counseling when the CHWs could provide their personal account and advise the families on whether the hardware was being used appropriately. Another study found that types of in-kind payment including material items such as backpacks, raincoats, home improvement equipment, educational materials sometimes allied with monetary incentives to motivate the CHWs’ job performance, but in the long run provision of incentives in too many forms proved unsuccessful and even demotivating [30].

Rural women with primary education and little to no experience are often selected by NGOs to work as CHWs. For this reason, training plays a vital role in their professional development. In the WASH Benefits study, CHWs received a concise but detailed training on technical health content as well as topics such as building rapport with the community, engaging in community dialog and problem solving, which are essential for work at the community level. Lessons that CHWs learned from these training sessions transformed ordinary community members into professional/social workers in the eyes of the community and boosted their level of confidence, skill and knowledge to motivate other people of the same community. Through supportive supervision and competency-based training, CHWs can feel that they are part of the health system [14]. Well trained health workers who have good supervision and receive regular payment can engage the community in health-related empowerment. Our CHWs conducted home visits and held group sessions to a much greater degree than in most CHW programs [31,32,33].

Because we conducted this assessment after three years of the intervention, CHWs had some difficulties recalling their experiences from the early days of the project but were more confident in describing recent experiences. Researchers in the future should conduct an assessment at two time points: an earlier or midterm stage which might help in the timely addressing of any arising issues and at the end stage to capture authentic longitudinal/trajectory data. The comparison would be able to show how issues and situations changed over the entire study period.

There are different categories of health workers in Bangladesh employed by both government and non-government entities. Health workers employed by the government include community health care providers (CHCP), family welfare assistants (FWA), health assistants (HA), health workers of the National Nutrition Program (NNP), and multipurpose health volunteers under the Ministry of Health and Family Welfare (MoH&FW). Non-government entities include BRAC’s Shastha Sabika, CHWs in research projects similar to WASH Benefits, and community promoters employed by other NGOs, all of which are similar in terms of qualifications, roles and responsibilities [34,35]. However, government recruited CHWs are paid according to the government pay scale which is higher, they receive more benefits and they have a longer job guarantee or a longer duration of project activities, in comparison to the CHWs in the WASH Benefits study. For these reasons, while the Government sets certain level criteria for new recruitments, they receive over-qualified applicants. In contrast, we had less qualified applicants than anticipated based on our call for applicants. Moreover, government health workers enjoy higher status in the community, whereas CHWs from WASH Benefits had to work harder to achieve that level of status and acceptance [36]. On the other hand, WASH Benefits CHWs were responsible for a smaller number of households than government or NGO CHWs and were able to develop closer relationships and provide more detailed follow up; the pay was better per hour compared with that of full-time employment offered by the Government, so their workload was more feasible. If other studies working with CHWs with similar responsibilities, training, incentives and supervision, adopt the approaches we describe in this paper, it is possible that the CHWs may perform their duties equally well or better.

## 5. Conclusions

There are many studies that seek to understand how CHWs engage in delivering health programs and devise methods to improve CHW attrition, retention and motivation. However, few studies examine the effects these health programs have on the social experience of the CHWs while serving in the health program [37,38,39,40,41]. Our study found that the lived experience of CHWs was multi-faceted, first influenced by their decision to become CHWs, and then by the skills and knowledge acquired on the job which enriched their social experience and along with the right materials supplied and inputs from their supervisors led them to feel successful. Family and community valuation were important contributors to a positive experience enhanced by the CHWs’ increased financial capability and influence in family decision making and helped them overcome initial challenges. Findings from this study suggest that high quality CHW training that includes the CHWs’ own modeling of targeted behaviors and technologies, provision of in-kind and cash payments to the CHWs, effective supervision, a manageable workload and longer project engagement can positively influence the experience of CHWs and can be used by future health programs to achieve high job satisfaction and retention of their CHWs. This can be especially important for women CHWs who may not have many other opportunities to engage in high value social activities. Evaluations of CHW programs should also consider the CHWs’ own lived experiences as important outcomes and take the effort to foster positive experiences among their CHWs.

## Figures and Tables

**Table 2:** Demographic characteristics of CHW study participants.

Total Number of CHWs in the Assessment	135
In-depth interview respondents	18
Focus groups participants	117
Mean age	29
	Frequency (%)
Marital status at the time of data collection	
Married	130 (96)
Divorced/separated	4 (3)
Unmarried	1 (0.7)
Years of education at the time of data collection	
8 years	22 (16)
9 years	23 (17)
10 years	68 (50)
12 years	22 (16)
Duration of working in the WASH Benefits study	
≤2 years	34 (25)
≤3 years	101 (75)

**Table 3:** Themes from the thematic analysis of the interviews and focus groups conducted with female CHWs.

Topic	Major Theme	Definition of the Themes
Anticipated benefits in becoming a CHW	(1) Importance of financial and other factors influencing decision to become a CHW	Explanations of financial and other issues considered before agreeing to work as a CHW
Benefits experienced while serving as a CHW	(2) Increased knowledge and skills	Explanations of the knowledge and skills earned during the period of working as a CHW
	(3) Family and community valuation	Explanations of what neighbors and relatives think about the CHW, how they treat the CHW and how they support/oppose the CHWs’ duties; Explanations of changes over time in community/neighbors’s thoughts
	(4) Importance of monetary and other incentives	Explanations of material benefits, opportunities to practice focus behaviors for the CHWs’ own good, and monetary rewards received while working as a CHW
Challenges connected with CHW service	(5) Overcoming anticipated and experienced challenges	Explanations of anticipated difficulties before becoming a CHW; Explanations of difficulties come across while working as a CHW; Explanations of how difficulties were overcome
Perceived profile of a CHW	(6) Perceived qualities of and other factors for a successful CHW	Explanations of qualities needed in a CHW to succeed in implementing a complex intervention among rural communities; Explanations of other factors contributing to CHW success

## Data Availability

The data that support the findings of this study are not publicly available because we did not ask participants to consent to raw data sharing outside of the research team. Public sharing of the data could compromise anonymity and research participant consent.
